# H19 and TUG1 lncRNAs as Novel Biomarkers for Irritable Bowel Syndrome in Diabetic Patients

**DOI:** 10.3390/biomedicines10112978

**Published:** 2022-11-19

**Authors:** Marwa M. Esawy, Noorah Saleh Al-Sowayan, Maysa A. Mobasher, Amir Abd-elhameed, Elsayed S. Abd elbaser, Shereen A. Baioumy, Marwa A. Shabana

**Affiliations:** 1 Clinical Pathology Department, Faculty of Human Medicine, Zagazig University, Zagazig 31527, Egypt; 2 Department of Biology, College of Science, Qassim University, Buraydah 52377, Saudi Arabia; 3 Pathology Department, Biochemistry Division, College of Medicine, Jouf University, Sakaka 72388, Saudi Arabia; 4 Internal Medicine Department, Faculty of Human Medicine, Zagazig University, Zagazig 31527, Egypt; 5 Tropical Medicine Department, Faculty of Human Medicine, Zagazig University, Zagazig 31527, Egypt; 6 Microbiology and Immunology Department, Faculty of Human Medicine, Zagazig University, Zagazig 31527, Egypt

**Keywords:** diabetes, irritable bowel syndrome, LncRNA H19, LncRNA TUG1, severity

## Abstract

Introduction: Irritable bowel syndrome (IBS) is a gastrointestinal disorder due to enteric nervous system impairment that produces different patterns of digestion. IBS is a common finding in diabetic patients. The functions of lncRNAs in IBS are still not clear and need to be further investigated. The aim of this study was to assess the diagnostic roles of lncRNA H19 and TUG1 for IBS associated with diabetes and to evaluate their association with clinical and laboratory findings. Subjects and Methods: Samples from 42 diabetic patients, 42 diabetic patients with IBS, and 42 healthy controls were obtained. The LncRNA H19 and TUG1 expressions were measured by quantitative real-time PCR. Results: The patients with IBS had significantly lower levels of lncRNA H19 and TUG1 expression than the healthy controls and diabetic-only patients (*p* < 0.001). LncRNA H19 and TUG1 can discriminate between diabetic-only patients and those with IBS (areas under the ROC curves of 0.95 and 0.722, respectively). The TUG1 expression levels were significantly different among types of IBS (IBS-D lower than IBS-M and IBS-C lower than IBS-M; *p* = 0.0165 and *p* = 0.043, respectively). H19 and TUG1 were downregulated in patients with poor glycemic control. lncRNA H19 and TUG1 expression in diabetic patients with IBS significantly negatively correlated with the IBS severity scoring system. Both lncRNAs’ expression significantly predicted the disease severity. LncRNA H19 expression can be an independent predictor for disease severity (adjusted odds ratio = 0.00001, 95% CI = 0–0.5, *p* = 0.045). Conclusions: Diabetic patients with IBS had significantly lower levels of lncRNA H19 and TUG1 expression than healthy controls and diabetic-only patients. LncRNA H19 had better diagnostic performance criteria for IBS. LncRNA H19 expression can be an independent predictor for IBS severity.

## 1. Introduction

Irritable bowel syndrome (IBS) is classified as a functional gastrointestinal impairment due to the absence of a known precise clinical cause, such as intestinal inflammation or infection. The most common clinical manifestations of IBS are frequent changes in intestinal habits, which are primarily defined by variations in stool consistency (constipation, diarrhea, or alteration). An additional symptom includes abdominal pain that is relieved after defecation [1].

IBS has several potential causes and risk factors, including age-related stress, female sex, a family history of the condition, an unbalanced diet, and a diet that is aggressive to the gastrointestinal tract. IBS is considered a functional gastrointestinal disorder with ROME IV criteria. IBS is now defined as a disorder of the gut–brain connection [2]. One of the candidate potential organic origins of the pathophysiology underlying IBS is the enteric nervous system (ENS), which produces different patterns of digestion. The ENS controls the muscular, neurohormonal, and secretory systems of the gastrointestinal tract [3].

Numerous alterations in gastrointestinal motility and related symptoms, including nausea, bloating, discomfort, diarrhea, and constipation, are linked to diabetes [4]. The ENS plays a crucial role in the pathophysiology of altered gastrointestinal functioning in diabetes, which is a complex process. The loss of inhibitory neurons in early diabetic enteric neuropathy is one of the changes in the inhibitory enteric neurons that are described [5]. Through alterations in the intestinal smooth muscle or variations in extrinsic neuronal regulation, diabetes can also impair gastrointestinal motility. The pathogenesis of these ENS changes includes the hyperglycemia and oxidative stress that occur in diabetes [6]. Hyperglycemia and related disorders cause severe destruction to various functions of the body, especially blood vessels and the nervous system [7].

In recent years, much research on long-chain noncoding RNAs (lncRNAs) has been published. However, the functions of lncRNAs in IBS are still not clear and need to be further investigated. lncRNA H19 plays a regulatory role in neurogenesis and the differentiation of neural stem cells during brain development or maturation [8]. Moreover, lncRNA H19 can regulate the intestinal mucosal mechanical barrier through various mechanisms and indirectly participate in the progression of intestinal diseases [9]. Downregulation of cell-free lncRNA H19 was observed in diabetes [10,11]. Diabetes severity has been associated with lower lncRNA H19 expression [12]. Reduced lncRNA H19 expression has been linked to diabetic clinicopathological abnormalities [12]. The onset and progression of IBS was shown to be significantly influenced by the downregulation of LncRNA H19 [13]. lncRNA taurine-upregulated gene 1 (TUG1) is actively involved in various physiological processes, including regulating genes in epigenetics and transcription [14]. It was discovered that the lncRNA TUG1 reduced the apoptosis and inflammatory response generated by the tumor necrosis factor, which has been linked to the etiology of IBS [15]. Intestinal epithelial cells are protected by lncRNA TUG1 from the damage caused by high glucose and high fat levels [16].

In fact, there are multiple IBS biomarker types that are intended to enhance diagnosis, distinguishing it from other organic diseases and differentiating amongst IBS subtypes. Currently, several potential biomarkers have been proposed (cytokines, serologic markers, and cellular/molecular markers) [17,18]. However, IBS biomarkers are unsatisfactory because of the small study populations and modest accuracy in excluding other organic disorders [9]. The full etiology and origin of the disease, as well as a detailed description of the molecular diagnostic biomarkers, are still unresolved issues [18]. The goal of this study was to assess lncRNA H19 and TUG1’s potential as diagnostic molecules for IBS and to evaluate their association with clinical and laboratory findings.

## 2. Materials and Methods

### 2.1. Study Design

The case-control method was used in this study. In Zagazig University Hospitals, from July 2022 to September 2022, samples from diabetic patients, diabetic patients with IBS, and healthy controls were obtained. The Faculty of Medicine Institutional Review Board approved this research (Zagazig University, No. ZU-IRB#9913). All patients understood and signed this study consent form prior to enrollment. The consent form included information about the purpose of the study, the sampling procedure, and participant rights.

### 2.2. Subjects

Based on a statistical power of 80% and a 95% confidence interval (CI), the sample size was determined. From a prior study, the mean difference and standard deviation were obtained [12]. The Epi Info software 6 was used to perform this calculation (Atlanta, GA, USA). The calculation of the sample size revealed 42 subjects in each group. Samples from 42 diabetic patients, 42 diabetic patients with IBS, and 42 healthy controls were obtained, all aged 18 years or more. By fulfilling the Rome IV criteria [19], IBS was diagnosed. A medical history was obtained along with abdominal imaging, blood, and/or fecal tests to rule out organic disease. The excluded patients were those who had associated psychological problems, chronic kidney disease, cancer, or inflammatory diseases. Additionally excluded were individuals who had undergone abdominal surgery within the previous six months. Patients were categorized into IBS subtypes based on their most common bowel habits: diarrhea (IBS-D), constipation (IBS-C), and mixed stool pattern (IBS-M). Patients with diabetes without gastrointestinal symptoms and healthy controls who were age- and sex-matched were both enrolled. To rule out the existence of any past or present gastrointestinal diseases or complaints, a brief medical history was collected.

Body mass index (BMI) was estimated using the equation [weight (kg)/height (m^2^)] [20].

### 2.3. Sampling

All individuals were asked to fast for 12 h. After an 8 h fast, whole blood was obtained in one plain tube, to measure the fasting glucose, and two EDTA tubes: the first tube for the HbA1c assay and the second for lncRNA expression. After the end of the 12 h fast, a further sample was taken in a plain tube for the evaluation of the lipid profile. Phlebotomists drew 2 mL blood in each tube for a total of 4 tubes for each patient (BD Vacutainer^®^, Becton Dickinson & Co, East Rutherford, NJ, USA). The plain tubes were collected, allowed to clot at room temperature for 30 min, and then centrifuged at 1200× *g* for 10 min to separate the serum. Centrifugation was used to separate the plasma for lncRNA expression (3000× *g*, 4 °C, 10 min).

### 2.4. Method

#### 2.4.1. Laboratory Tests

The lipid profile, including total cholesterol, triglycerides, high-density lipoprotein cholesterol (HDL-C), and low-density lipoprotein cholesterol (LDL-C), as well as the fasting blood sugar were all measured in the laboratory on a Cobas 8000-C702 Modular Analyzer (Roche, Germany). HbA1C was determined using the Roche, Germany-based Cobas 6000-C501 Modular Analyzer.

#### 2.4.2. LncRNA Expression

Using an miRNeasy Serum/Plasma Kit (QIAGEN, GmbH, Hilden, Germany), all RNAs were isolated from the plasma, following the manufacturer’s instructions. Each reverse transcription reaction contained one μg RNA; RNA quantity and quality were assessed by a NanoDrop-2000 spectrophotometer (Thermo Scientific, Waltham, MA, USA), and the cDNA was produced using the miScript RT II kit in accordance with the manufacturer’s instructions (QIAGEN GmbH, Hilden, Germany).

The miScript SYBR Green PCR kit was used on a StepOne™ System real-time PCR instrument (Applied Biosystems, Foster City, CA, USA), and quantitative real-time PCR (RT-qPCR) was carried out to determine the expression levels of distinct lncRNAs. The reaction system was in a 20 µL volume. The RT-qPCR thermal protocol was set as follows: 95 °C for 10 min; then, 40 amplification cycles of 60 °C for 30 sec and 72 °C for 1 min. Annealing curve analysis was performed to confirm the specificity of the reaction.

The primer sequence data were as follows: H19 forward primer: 5’-TACAACCACTGCACTACCTG-3’; H19 reverse primer: 5’-TGGAATGCTTGAAGGCTGCT-3’; TUG1 forward primer: 5’-TAGCAGTTCCCCAATCCTTG-3; TUG1 reverse primer: 5’-CACAAATTCCCATTCCC-3; GAPDH forward primer: 5’-AC CAGGAAATGAGCTTGACA- 3; GAPDH reverse primer: 5’-GACCACAGTCCATGCCATC-3. GAPDH was utilized to normalize the results because its expression level has been established to be high and constant in many different cells and tissues [21]. The expressions were normalized using the 2-ΔΔCT calculation, and the relative expression of these lncRNAs was estimated.

### 2.5. Statistical Analysis

The research parameters displayed a non-normally distributed pattern by the Shapiro–Wilk test. For the comparison of multiple parameters, the Kruskal–Wallis H test, and the post hoc test (Dunn’s test) were used. When relevant, the Mann–Whitney U test and the chi-squared test were applied. The receiver operating characteristic (ROC) curve is used to assess a biomarker’s potential to distinguish disease status. The diagnostic efficacy was studied using the area under the ROC curve (AUC) and its 95% confidence interval (CI). Youden’s index measures a biomarker’s potential efficacy; the highest Youden’s index was used to determine the ideal cutoff point. Spearman’s correlation coefficient was used to determine the relationship between the expression of lncRNAs and disease features. To evaluate the risk, the odds ratio was determined using univariate and multivariate logistic regression analysis. The statistical software utilized in this investigation was SPSS 20.0 (Chicago, IL, USA), and significance was determined at a *p*-value <0.05.

## 3. Results

The participants’ clinical, demographic, and laboratory information is shown in Table 1. The results demonstrated that the body mass index (BMI) was significantly higher in diabetic patients and diabetic patients with IBS than in the controls (*p* < 0.001 and 0.012, respectively). The most common type of IBS was the IBS-D (57.1% of patients). The lipid profile, except for LDL, showed higher levels in diabetic patients with IBS when compared to diabetic-only patients (*p* < 0.001). Diabetic patients with IBS had higher cholesterol and LDL-C in comparison to the healthy controls (*p* < 0.001).

**Figure 1:**
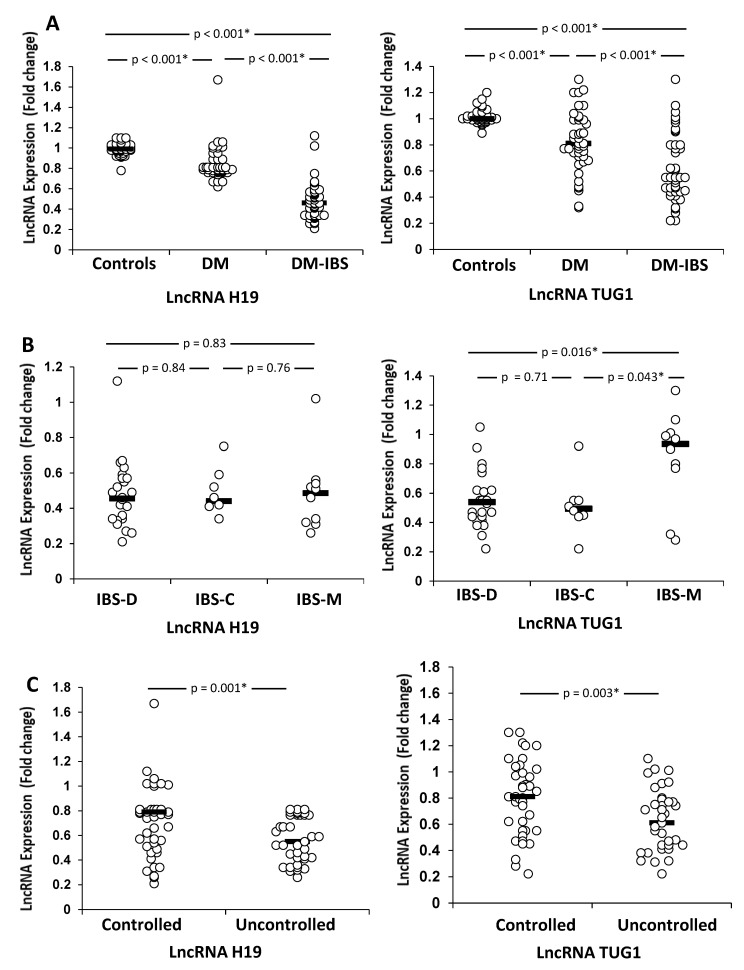
Dot plot of H19 and TUG1 expression in (**A**) healthy controls and different patient groups; (**B**) according to IBS type; (**C**) according to diabetes control measured by HbA1C. The horizontal bars show the medians. A and B: Kruskal–Wallis test followed by Dunn’s multiple comparisons test were used for statistical analysis. C: Mann–Whitney U test was used for statistical analysis. * Significant.

The findings showed that the diabetic patients with IBS had significantly lower levels of lncRNA H19 and TUG1 expression than the healthy controls and diabetic-only patients (*p*< 0.001; Figure 1A). Additionally, the lncRNA TUG1 expression levels were significantly different among types of IBS (IBS-D lower than IBS-M and IBS-C lower than IBS-M; *p* = 0.0165 and *p* = 0.043, respectively) (Figure 1B). Moreover, the differences between diabetics with good glucose control (7% HbA1c) and those with poor control (≥7% HbA1c) in terms of lncRNA expression were evaluated. The expression of H19 and TUG1 was downregulated in patients with poor glycemic control compared to good glucose control (*p* = 0.001 and *p* = 0.003, respectively) (Figure 1C).

**Figure 2:**
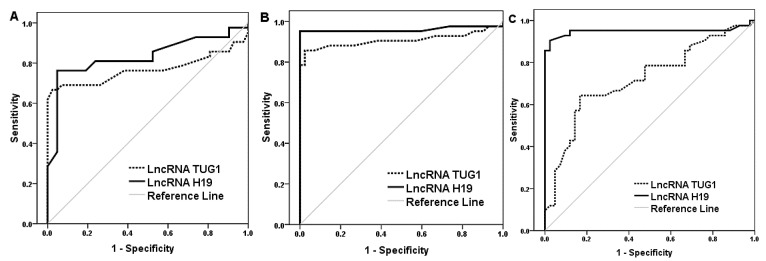
ROC curve for lncRNA expression. (**A**) Differentiation between healthy controls and diabetes, (**B**) differentiation between healthy controls and IBS diabetic patients, (**C**) differentiation between diabetic patients and diabetic patients with IBS.

Furthermore, an ROC curve analysis was performed. The areas under the ROC curves of lncRNA H19 and TUG1 expression in diabetic patients and healthy controls were 0.832 and 0.768, respectively (Figure 2A). The lncRNA H19 and TUG1 expression had areas under the ROC curves for differentiation between the healthy controls and diabetic patients with IBS of 0.96 and 0.908, respectively (Figure 2B). Using lncRNA H19 and TUG1 for discrimination between diabetic-only patients and diabetic patients with IBS showed areas under the ROC curves of 0.95 and 0.722, respectively (Figure 2C). Overall, lncRNA H19 showed higher performance characteristics (Table 2).

Table 3 shows that the expression of lncRNA H19 in the diabetes group correlated negatively with HbA1c, cholesterol, triglycerides, and LDL-C, whereas lncRNA TUG1 only correlated negatively with HbA1C. lncRNA H19 and TUG1 expression in diabetic patients with IBS had significant negative correlations with IBS-SSS.

The logistic regression assessed the ability of lncRNA to predict the disease severity by differentiating mild and moderate cases from severe ones. Both lncRNAs’ expression significantly predicted the disease severity. Some variables showed some association but with no significant value. In the multivariate analysis for all the variables listed in Table 4, the lncRNA H19 expression was the only one still significantly associated with the disease severity. The low lncRNA H19 expression was associated with the severe group (AOR = 0.00001, 95% CI = 0–0.5, *p* = 0.045).

## 4. Discussion

The prevalence of diabetes and its related complications are increasing significantly globally [22]. Diabetes and IBS are both common conditions. The two conditions may be related. Small intestinal and colorectal dysfunctions are common in patients with longstanding diabetes, especially in those with gastroparesis. Diabetes-related enteropathy may present with diarrhea, constipation, or fecal incontinence. The mechanism of the development of enteropathy is similar to that of the upper gastrointestinal involvement in diabetes. Advanced glycation end products cause damage to cellular DNA and tissues in diabetes. Damage to the myenteric nerve because of autonomic neuropathy and fibrosis of the intestinal muscular layers cause stasis of the intestinal contents. Reduced bowel motility results in constipation, which may sometimes lead to overflow incontinence. Small intestinal bacterial overgrowth, which can result in diarrhea, is usually a consequence of intestinal stasis [23].

The absence of accurate biological markers continues to be a major feature of IBS. This study was conducted to better understand the roles performed by lncRNA H19 and TUG1 as diagnostic markers for IBS in diabetic patients, as well as to assess their relationships to clinical and laboratory data. This study showed that IBS-D was the most typical form of the disease. These results corroborated those of Kibune-Nagasako et al. [24], who found that IBS-D was the most common IBS subtype, followed by IBS-C and IBS-M.

The findings of this study showed that compared to the controls, diabetic patients and diabetic patients with IBS had significantly higher body mass indices (BMI). The BMI determined that 29% of the IBS patients were obese, and 31% were overweight [24]. Additionally, Javadekar et al. [25] reported that patients with IBS had decreased HDL-C and higher values for weight, waist circumference, BMI, and fasting glucose. Similar to the findings of Gulcan et al. [26], it was found that IBS was significantly associated with an elevated fasting glucose level. On the other hand, Andalib et al. [27] failed to detect evidence to suggest a link between type 2 diabetes mellitus and IBS. Their investigation found no correlation between IBS and type 2 diabetes mellitus or body mass index. Visceral abdominal obesity has already been linked to an increased risk of IBS [28]. This discrepancy’s likely cause is the high proportion of black women as subjects that Andalib et al. [27] included. The racial difference in the population under study may be the cause of that variation.

This study found that patients with IBS who were diabetic displayed high levels of lipids, except for HDL-C. Patients with diabetes and diabetic patients with IBS had significantly higher serum levels of total cholesterol, triglycerides, and LDL-C cholesterol than normal values. [24,28].

Non-protein-coding RNA molecules longer than 200 nucleotides are known as LncRNAs. Many lncRNAs have different functional roles in diseases, including epigenetic, transcriptional, and posttranscriptional control, even though their mechanisms are not entirely understood [29]. One of the first recognized lncRNAs linked to human disease was H19, and another was TUG1. LncRNA TUG1 plays a crucial role in several human disorders, including osteoporosis and cardiomyocyte ischemia, and recent studies have shown that it may also play a role in the development of many malignancies [30]. Apoptosis and angiogenesis are two biological processes that LncRNA H19 encourages [31]. Numerous illnesses, including cancer, type 2 diabetes, and hypertrophic cardiomyopathy, are linked to the abnormal expression of H19 [32].

More and more data point to the possibility that hyperglycemia might potentially cause epigenetic changes that promote oxidative stress in addition to biochemical mechanisms [33]. We may obtain a new understanding of the pathophysiology of diabetes mellitus and related complications through epigenetic mechanisms, which could lead to the development of new treatment targets. Noncoding RNAs, among other epigenetic variables, have been linked to the etiology of IBS [34].

Diabetes and IBS were found to have altered lncRNA expression [35]. This study showed that diabetic patients with IBS had considerably lower levels of lncRNA H19 expression than healthy controls and diabetic-only patients. Additionally, patients with poor glycemic control had lower levels of H19 expression than those with adequate glucose control. This was also demonstrated in an animal model where diabetic mice had lower levels of H19, which increased hepatic gluconeogenesis and glucose production [36]. However, Tello-Flores [37] showed that diabetic patients with poor glycemic control had a considerable rise in LncRNA H19 levels. In diabetic patients with IBS, low levels of lncRNA H19 and TUG1 expression were observed to have a strong negative connection with the IBS severity score. Similarly, Chao et al. [13] reported that the relative expression of LncRNA H19 in the colonic tissues of IBS-D patients was reduced significantly.

The significance of LncRNA H19 in intestinal inflammation is receiving more and more attention. LncRNA H19 and intestinal barrier dysfunction are related, according to Chen et al.’s investigation of ulcerative colitis [38]. Therefore, LncRNA H19 and the permeability of the intestinal mucosa brought on by barrier dysfunction may be closely related. Overall, it is fair to assume that intestinal barrier dysfunction is related to the lower expression of LncRNA H19 in the intestinal mucosa of IBS-D patients [39]. Elevation of lncRNA H19 is involved in regulating the epithelial barrier functions [40]. Expression of LncRNA H19 in the intestinal mucosa of IBS-D patients was significantly decreased, resulting in the inflammatory factor secretion, with a molecular change peculiar to barrier dysfunction [41]. LncRNA H19 correlated to the change in the intestinal barrier function in IBS-D patients [13]. However, LncRNA H19 was increased in diabetes patients from certain studies in a comprehensive review by Dieter et al. [42] but downregulated in other studies, which might be addressed by variations in the types of samples investigated (serum, pancreatic islets cells, liver cells, and peripheral blood mononuclear cells). Characterizing lncRNA expression is the first step toward elucidating lncRNAs’ molecular mechanisms, which will provide further comprehensive insights into pathogenesis and ultimately lead to novel therapeutic strategies [43].

An obvious strength of this study was the selection of lncRNAs as markers for IBS. LncRNAs have been investigated as promising diagnostic and therapeutic markers in different diseases due to their stability and easy detection in biological fluids. Limitations still exist in the present study, including the lack of in vivo and in vitro exploration of related mechanisms, the signaling pathways related to lncRNA H19-induced intestinal dysfunction, and the requirement for further evaluations to generalize the results.

## 5. Conclusions

This study assessed lncRNA H19 and TUG1 expression by qRT-PCR in diabetic patients with IBS. The lncRNA H19 and TUG1 expressions had significantly lower levels in diabetic patients with IBS than healthy controls and diabetic-only patients. LncRNA H19 had better diagnostic performance criteria for IBS. LncRNA H19 expression can be an independent predictor for IBS severity. Large-scale studies are needed to validate our results and to investigate other lncRNA candidates in IBS.

## Figures and Tables

**Table 1:** Demographic, clinical, and biochemical characteristics of the studied groups.

Parameter	Control Group (No. = 42)	Diabetes Group (No. = 42)	Diabetic IBS Group (No. = 42)	*p*
Age (years)	36 (21–60)	37.5 (22–5)	36 (22–56)	0.35
Sex Male/female	20/22 (47.6/52.4)	22/20 (52.4/47.6)	17/25 (40.5/59.5)	0.75
Duration of IBS symptoms (months)	-------	------	7 [1–30]	
Smoking	20 (47.6)	20 (47.6)	26 (61.9)	0.32
BMI (kg/m^2^)	25.4 (23.3–29.5)	27 (23.3–29.6) a	27.4 (22.6–33.2) a	0.002 *
IBS Type:				
IBS—diarrhea			24 (57.1)	
IBS—constipation			8 (19)	
IBS—mixed			10 (23.9)	
Severity:				
IBS-SSS			289 (80–430)	
Mild			6 (14.3)	
Moderate			17 (40.5)	
Severe			19 (45.2)	
Laboratory Tests:				
Fasting glucose (mg/dL)	83.4 (74.8–101)	120.3 (108.3–224.3) a	132.3 (98–313.6) a	<0.001 *
HbA1c (%)	4.1 (3.2–5)	6.1 (4.9–8.9) a	7.2 (5.5–15.2) a, b	<0.001 *
Total cholesterol (mg/dL)	127.9 (76.4–188)	117 (88–171.3)	160.8 (100.9–289.5) a, b	<0.001 *
Triglyceride (mg/dL)	82 (65.3–145.6)	84 (74–124.2)	98.6 (72.4–199.4) b	0.036 *
HDL-C (mg/dL)	42.7 (32.1–62.3)	42.1 (26.9–55.2)	42.4 (29.8–57.4)	0.78
LDL -C(mg/dL)	61.7 (26–122.5)	63.8 (23.2–115.9)	101.5 (41.9–224.6) a, b	<0.001 *

No.: number of subjects; IBS: irritable bowel syndrome; IBS-SSS: IBS severity scoring system; BMI: body mass index; HbA1c: hemoglobin A1c; HDL-C: high-density lipoprotein cholesterol; LDL-C: low-density lipoprotein cholesterol. Data are presented as No. (%) or median [min-max]. *p*: significance of Kruskal–Wallis H test then post hoc test (Dunn’s test); a: the significant difference in comparison to the control group; b: the significant difference in comparison to the diabetes group. * Significant.

**Table 2:** The diagnostic performance characteristics of the studied markers.

Marker	AUC [95% CI]	Cutoff	Youden’s Index	Sensitivity (%)	Specificity (%)	PPV (%)	NPV (%)	Accuracy (%)
Differentiation between healthy controls and diabetic-only patients								
LncRNA H19	0.832 [0.737–0.927]	0.9	0.71	76.2	95.2	94.1	80	85.7
LncRNA TUG1	0.768 [0.653–0.882]	0.92	0.64	66.6	97.6	96.6	74.5	82.1
Differentiation between healthy controls and diabetic patients with IBS								
LncRNA H19	0.960 [0.905–1.015]	0.76	0.95	95.2	100	100	95.5	97.6
LncRNA TUG1	0.908 [0.830–0.985]	0.94	0.83	85.7	97.6	97.3	87.2	91.7
Differentiation between diabetic patients and diabetic patients with IBS								
LncRNA H19	0.950 [0.889–1.011]	0.66	0.88	90.5	97.6	97.4	91.1	94
LncRNA TUG1	0.722 [0.611–0.833]	0.64	0.47	64.3	83.3	79.4	70	73.8

AUC: area under the ROC curve; CI: confidence interval; PPV: positive predictive value; NPV: negative predictive value.

**Table 3:** Association of lncRNAs, clinical data, and laboratory data in the studied patients.

Parameter	LncRNA H19				LncRNA TUG1			
	Diabetes Group (No. = 42)		Diabetic IBS Group (No. = 42)		Diabetes Group (no. = 42)		Diabetic IBS Group (no. = 42)	
	r	*p*	r	*p*	r	*p*	r	*p*
Age	−0.13	0.4	−0.02	0.88	−0.1	0.52	−0.3	0.05
BMI	−0.25	0.11	0.3	0.85	−0.27	0.09	0.19	0.22
Symptoms duration	----	----	0.05	0.73	----	----	0.16	0.31
IBS-SSS	----	----	−0.46	0.002 *	----	----	−0.37	0.017 *
Fasting glucose	0.07	0.66	0.24	0.12	0.04	081	0.23	0.14
HbA1c	−0.39	0.01 *	−0.28	0.07	−0.31	0.04 *	−0.07	0.66
Cholesterol	−0.42	0.006 *	0.19	0.91	0.09	0.57	0.01	0.98
LDL-C	−0.46	0.002 *	0.05	0.75	0.07	0.66	−0.01	0.93
Triglycerides	−0.36	0.02 *	−0.24	0.13	0.02	0.9	−0.16	0.33
HDL-C	0.18	0.24	0.14	0.37	−0.11	0.5	0.21	0.85
LncRNA H19	1		1		0.13	0.4	0.24	0.12
LncRNA TUG1	0.13	0.4	0.24	0.12	1		1	

r: correlation coefficient; IBS: irritable bowel syndrome; IBS-SSS: IBS severity scoring system; BMI: body mass index; HbA1c: hemoglobin A1c; HDL-C: high-density lipoprotein cholesterol; LDL-C: low-density lipoprotein cholesterol. * Significant.

**Table 4:** Logistic regression analysis of risk factors for IBS severity.

Covariate	Univariate Analysis		Multivariate Analysis	
	OR (95% CI)	*p*	AOR (95% CI)	*p*
Age	1.1 (0.99–1.24)	0.07	1.53 (0.91–2.58)	0.11
Sex (male)	0.33 (0.09–1.21)	0.09	0.42 (0.01–20.6)	0.66
Smoking	1.1 (0.32–3.8)	0.89	6.39 (0–699)	0.63
Symptoms duration	1.02 (0.95–1.09)	0.58	1.04 (0.81–1.35)	0.75
BMI	0.73 (0.53–1.01)	0.05	0.35 (0.07–1.6)	0.18
Fasting glucose	1 (0.99–1.01)	0.49	1.03 (0.98–1.08)	0.17
Diabetes control	0.69 (0.2–2.35)	0.55	0.06(0–406)	0.63
Total cholesterol	0.99 (0.98–1)	0.38	0.02 (0–7.4)	0.14
Triglyceride	1.02 (0.99–1.06)	0.19	2.2 (0.79–6.5)	0.13
HDL-C	0.98 (0.9–1.07)	0.68	381 (0.13–1089)	0.14
LDL -C	0.99 (0.98–1.1)	0.39	452 (0.12–1602)	0.14
LncRNA H19	0.001 (0–0.33)	0.019 *	0.00001 (0–0.5)	0.045 *
LncRNA TUG1	0.02 (0.001–0.45)	0.01 *	0.01(0–8.3)	0.13

OR: odds ratio; AOR: adjusted OR; BMI: body mass index; HbA1c: hemoglobin A1c; HDL-C: high-density lipoprotein cholesterol; LDL-C: low-density lipoprotein cholesterol *: significant.

## Data Availability

Data will be available on request.
